# Advancements and obstacles in acrylamide detection and mitigation in food products

**DOI:** 10.1016/j.fochx.2025.102593

**Published:** 2025-05-27

**Authors:** Ancuţa-Veronica Lupăescu, Mircea Oroian

**Affiliations:** a Faculty of Medicine and Biological Sciences, “Ştefan cel Mare” University of Suceava, 13th University Street, Suceava 720229, Romania; b Suceava-Botoșani Regional Innovative Bioeconomy Cluster Association, Suceava 720229, Romania; c Faculty of Food Engineering, “Ştefan cel Mare” University of Suceava, 13th University Street, Suceava 720229, Romania

**Keywords:** Acrylamide, Food, Chromatography, Mitigation strategies

## Abstract

Acrylamide, a water-soluble compound formed naturally in foods during high-temperature cooking, poses significant health risks due to its carcinogenic and neurotoxic effects. This review offers a comprehensive analysis of advanced detection methods and mitigation strategies, underscoring how detection sensitivity influences the evaluation and optimization of acrylamide reduction strategies. Advanced chromatographic techniques, such as LC-MS/MS and GC–MS, enable trace-level quantification of acrylamide and serve as essential tools for validating the efficacy of mitigation strategies, including enzymatic, fermentation-based, and antioxidant treatments. Additionally, emerging biotechnological approaches, including engineered enzymes, probiotic detoxification, and nanostructured hydrocolloid systems, are discussed for their potential to minimize acrylamide while preserving sensory attributes. Limitations in current methodologies, including variability across food matrices and scalability challenges, are critically assessed. This study highlights recent progress in chromatographic methods for acrylamide detection and explores mitigation strategies to reduce its levels in heat-treated foods, emphasizing the importance of continued research in food safety.

## Highlights

• LC-MS/MS enables accurate trace acrylamide detection across complex food matrices.
• Enzyme engineering improves L-asparaginase stability for industrial mitigation.
• Fermentation and probiotics reduce acrylamide through precursor depletion mechanisms.
• Hydrocolloids and antioxidants impact both acrylamide and product quality.
• High analytical costs highlight the need for more accessible detection methods.

## 1 Introduction

Acrylamide, often known as acrylic amide, is a chemical compound with the molecular formula C₃H₅NO. Due to its chemical structure (CH_2_ = CHC(*O*)NH_2_), the substance exhibits a crystalline structure, highly soluble in water, with a white color that lacks any discernible odor. Acrylamide finds its predominant application in the manufacturing of polymers and various industrial uses (Taeymans et al., 2004). Moreover, it has the potential to arise naturally in specific foods during cooking procedures.

**Scheme 1:**
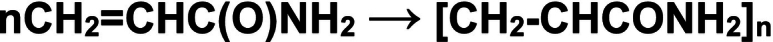
General polymerization reaction of acrylamide

Within the industrial context, acrylamide finds widespread applications in various industrial sectors playing a significant role in the production of polyacrylamide (PAM) polymers (See Scheme 1). This versatility is further amplified through the fabrication of copolymers, wherein acrylamide engages in polymerization alongside other monomers such as acrylic acid, resulting an anionic polyacrylamide commonly used in water treatment (Tepe & Çebi, 2019). Despite its widespread use, PAM may raise environmental concerns due to its persistence and potential release of acrylamide. Although PAM itself has low toxicity, its slow degradation is of concern (Gaytán, Burelo, & Loza-Tavera, 2021). Natural and human-induced factors contribute to the degradation of PAM, and while UV irradiation and thermal stress can release minimal acrylamide, incomplete polymerization during production is the main source of acrylamide release (Buczek, Cope, McLaughlin, & Kwak, 2017). Therefore, exposure to acrylamide can result not only from food cooking methods but also from consuming products that have come into contact with improperly stored PAM-based materials.

nCH_2_ = CHC(*O*)NH_2_ → [CH_2_-CHCONH_2_]_n_.

The health effects of acrylamide have garnered significant attention due to its potential risks to human health (Fan et al., 2023; Sarion et al., 2021). Acrylamide is classified as a toxic substance displaying potential carcinogenicity, neurotoxic effects, genetic damage and adverse impacts on reproduction and development (Curtis & Halford, 2016). It also possesses a notable ability to permeate biological barriers (Mesías & Morales, 2016). Acrylamide is naturally formed in certain foods during high-temperature cooking through a complex process involving the Maillard reaction between amino acids and reducing sugars. At temperatures above 120 °C (248 °F), amino acids like asparagine in food interact with reducing sugars such as glucose and fructose, triggering the Maillard reaction (Becalski et al., 2003). This leads to the creation of Amadori products—compounds that serve as intermediates. These products can further transform through reactions like dehydration and fragmentation, resulting in substances like 3-aminopropionamide and acrolein (Anese & Suman, 2013). Acrylamide, a concerning compound, emerges from acrolein's reactions, particularly its dehydration and decarboxylation. The formation of acrylamide depends on factors like cooking conditions, moisture, and precursor presence, and though the Maillard reaction that is a key contributor. However, other pathways might be at play in specific foods.

**Fig. 1:**
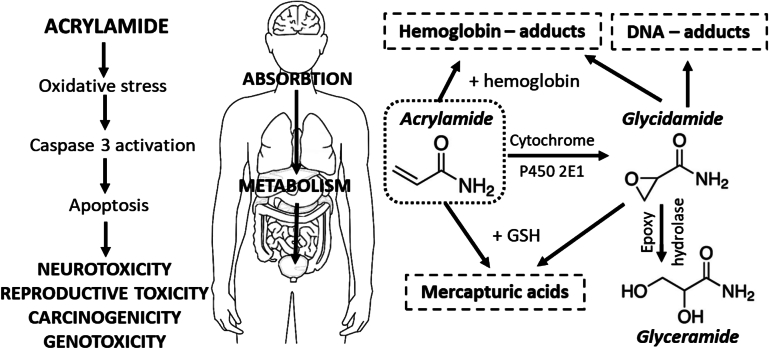
Representation of acrylamide-induced toxicity and its potential mechanism.

Following absorption through the gastrointestinal tracts of humans and animals, acrylamide (AA) undergoes passive diffusion throughout the entire body, generating biomarkers (See Fig. 1). Upon interaction with glutathione S-transferases, AA forms a conjugation with glutathione, subsequently metabolized into mercapturic acids, which are excreted in urine (Tiwary, Das, & Paul, 2022). This pathway primarily facilitates AA detoxification. However, glutathione consumption diminishes antioxidant levels, resulting in the accumulation of excessive active oxygen and inducing oxidative stress and neurotoxicity. Under metabolic transformation, AA is enzymatically converted into glycidamide by the cytochrome P450 enzyme system, particularly CYP2E1 (Ghamdi et al., 2020). Glycidamide exhibits the ability to bind with purine bases on DNA molecules, forming DNA adducts. This process inhibits neurotransmitter release, induces nerve terminal degeneration, damages nerve structures, and manifests distinct cumulative effects. Similar to AA, glycidamide can also form hemoglobin (Hb) adducts or undergo biotransformation until it forms mercapturic acids. Finally, glycidamide can undergo further hydrolysis to glyceramide by epoxide hydrolase which is also excreted in urine (Zhao, Zhang, & Deng, 2022)

Given its classification as a potential human carcinogen by international health organizations, the accurate analysis of acrylamide is imperative to evaluate exposure levels and counteract health hazards. Moreover, common foods like potatoes, bread, coffee, and snacks can exhibit varying acrylamide concentrations, making precise analysis essential for regulatory compliance and consumer safety (Timmermann et al., 2021). This importance is underscored by the fact that numerous countries have established acrylamide limits in food items, underscoring the necessity of dependable analysis to uphold safety standards and evade legal repercussions. As a result, precise acrylamide analysis aids health authorities in assessing risks associated with dietary habits and devising effective risk management and public health strategies (Biedermann et al., 2010). Consequently, manufacturers can enhance food quality and safety by employing acrylamide analysis for quality control and refining cooking techniques (Ledbetter et al., 2020). Additionally, comprehending the mechanisms and levels of acrylamide formation through research facilitates the creation of strategies to minimize its presence during food processing. This awareness also empowers consumers to make well-informed choices and facilitates international food trade through compliance with safety benchmarks.

In recent years, chromatographic techniques have become essential for the analysis of acrylamide, contributing significantly to the increase in sensitivity, efficiency of detection of this complex in matrices. Despite recent advances, existing methods face challenges related to the complexity of the food matrix and high operational costs. Innovations such as simplified derivatization, solid phase extraction (SPE) and advanced purification techniques have allowed for lower detection limits and supported more reliable analysis in practice. Also, new technologies such as nanotechnology-based biosensors allow the detection of acrylamide at extremely low concentrations, providing more affordable solutions for small food producers. These advances not only improve the accuracy of the analysis, but also transform current practices in the field.

Unlike many previous studies that examine acrylamide detection and mitigation strategies as separate research areas, the present manuscript takes an integrative approach, highlighting how the sensitivity and specificity of analytical techniques, particularly LC-MS/MS and GC-based methods, can directly support the evaluation and optimization of reduction strategies, such as enzymatic degradation or antioxidant application. This integrated perspective allows for the identification of specific trade-offs, such as the correlation between detection thresholds and potential changes in sensory properties, thereby facilitating the development of effective and practical solutions for acrylamide minimization in food products.

This review details the latest innovations in chromatographic detection technologies and explores emerging techniques for the reduction of acrylamide in food products. It also explores the integration of sustainable and affordable methods that not only minimize risks to human health, but also contribute to the production of high-quality food products without compromising nutritional value or flavor. In this context, significant progress in the development of innovative and safe solutions for industry, with the potential for widespread application, is highlighted. Furthermore, recent research suggests that, in addition to the Maillard reaction, other factors, such as preservatives and high levels of asparagine in raw materials, may play a significant role in the formation of acrylamide. These findings may open new directions in food processing, providing alternatives that reduce consumer exposure to this contaminant. As acrylamide poses important health risks, including carcinogenicity and neurotoxicity, the implementation of effective methods for its reduction becomes crucial to protect consumers and meet international standards. Thus, by examining recent advancements, the article aims to elucidate ongoing efforts to improve the safety and quality of heat-treated foods through efficient acrylamide analysis, while supporting the development of innovative processing strategies that minimize acrylamide formation, thereby protecting public health and helping the food industry comply with safety regulations.

## 2 Extraction procedures based on sorbent materials

Effective sample preparation and extraction are pivotal in acrylamide analysis due to the complexity of food matrices and the need to ensure accurate quantification. The process involves isolating acrylamide from the food matrix, concentrating it, and eliminating interfering compounds to enhance the sensitivity and reliability of the analysis. While there isn't a universally accepted extraction method for acrylamide from diverse food samples, considerable endeavors have been dedicated to crafting optimal extraction procedures that emphasize effective chromatographic separation and MS detection parameters (Norouzi et al., 2018).

The efficiency of extracting acrylamide from food matrices is impacted by a range of factors including particle size, defatting, choice of solvent, solvent-to-sample ratio, homogenization, mechanical forces, temperature and extraction time. Inaccuracies in analysis can arise from inadequate extraction caused by insufficient maceration, brief extraction durations or low temperatures, particularly when removing fat or evaporating the extractant. The purification techniques discussed in the literature often involve multi-step processes. In the standard initial treatment, samples are subjected to de-fatting using non-polar solvents. Additionally, the elimination of protein precipitates from solutions is accomplished through the application of Carrez solutions (Arisseto et al., 2008). Challenges in acrylamide extraction encompass its generation during procedures like methanolic Soxhlet extraction, labware contamination, and thermal degradation, with some foods potentially yielding higher acrylamide amounts under strongly alkaline conditions (pH 12) compared to normal conditions (pH 6) (Elbashir et al., 2014). Alternatively, employing acetonitrile reduces the co-extraction of typical matrix elements such as highly non-polar fats and assists in precipitating proteins (Petrarca et al., 2017). Moreover, acidified acetonitrile has showcased superior efficacy in extracting acrylamide across diverse food samples (Tölgyesi & Sharma, 2020).

In addition to the extraction solvent, the chosen extraction method also holds significant importance. As exemplified in a study involving the analysis of bread samples, the incorporation of ultrasonic treatment increased yield during extraction by assuring a proper homogenization of solid sample (Norouzi et al., 2018). Further refinement of extracts can be achieved through the application of solid-phase extraction (SPE) techniques, employing either normal-phase or reversed-phase beds. Moreover, the integration of ion-exchange sorbents and the adoption of the “QuEChERS” (Quick, Easy, Cheap, Effective, Rugged and Safe)-based approach, commonly employed in the analysis of pesticide residues, have been employed for the determination of acrylamide (Roszko et al., 2020).

Solid-phase extraction stands as a sample preparation technique widely employed in analytical chemistry to selectively isolate and concentrate target compounds from complex matrices before their analysis. In the SPE process, a solid-phase sorbent material is used to selectively retain the analytes while allowing unwanted matrix components to be washed away (Pavkovich & Bell, 2018). This method offers versatility and adaptability, allowing it to be applied to a diverse range of sample types, including environmental, food, pharmaceutical, and biological samples (Badawy et al., 2022).

The development of tailored extraction procedures for the selective extraction of acrylamide is based on the use of absorbent materials with tailored surface properties. Notably, multiwalled carbon nanotubes materials present an intriguing avenue for the selective extraction of acrylamide. For instance, chitosan-grafted multiwalled carbon nanotubes exhibited remarkable adsorption capabilities attributed to their unique structural characteristics, such as hollow and layered nanostructures and a substantial specific surface area (Zhao et al., 2015). These distinct features establish them as promising candidates for the efficient extraction of acrylamide from diverse matrices. Similarly, alumina, a pivotal sorbent material, holds remarkable significance in the analysis of acrylamide. Its intricate composition and structural attributes prove advantageous when dealing with intricate sample matrices, thus enabling it to play a critical role in achieving the effective and precise extraction of acrylamide. This is notably achieved through a simplified extraction mixture consisting of acetonitrile:water:formic acid, combined with the efficacy of dispersive solid-phase extraction (Prata et al., 2023).

The incorporation of various distinct sorbents within a single solid-phase extraction (SPE) step offers clear benefits in efficiently mitigating substantial extract interferences. Specifically, the inclusion of a mixed-phase SPE column containing C-18, strong cation, and anion exchange phases demonstrated its efficacy in eliminating compounds like melanoidins, trigonelline, chlorogenic acids, and caffeine from the coffee extract (Badawy et al., 2022). Consequently, this approach led to an enhanced recovery of acrylamide within the sample.

Despite their advantages, sorbent-based extraction methods for acrylamide analysis still face challenges related to matrix complexity, method standardization, and potential interference. Factors such as cost, co-extractives, variability, universality, and cross-contamination risk also impact results. Thus, researchers should select sorbents wisely, optimize conditions, and implement validation measures to ensure reliable outcomes.

## 3 Advanced chromatographic approaches

The development and validation of sensitive, accurate, and cost-effective analytical methods for detecting acrylamide in various food matrices at low μg/kg levels are essential for laboratories worldwide. Identifying a rapid and reliable extraction method, combined with an economical quantification technique, is crucial for incorporating acrylamide analysis into routine testing protocols. Since AA lacks fluorescence activity, most researchers utilize gas and liquid chromatography for its instrumental analysis.

Due to its structural properties, mass spectrometry in positive ion mode is the most effective tool for detecting acrylamide in food matrices at ppb levels (Negoiță et al., 2021). ESI is favored for its reliability and soft ionization, which preserves the precursor's original structure, ensuring high sensitivity for small molecules like acrylamide. However, accurate separation of acrylamide from co-extractive compounds remains challenging due to its low molecular mass, high polarity, and reactivity, even with MS/MS detection (Surma et al., 2017). Therefore, emphasis on clean-up and purification steps is essential for high method accuracy.

### 3.1 Gas chromatographic methods

With the advancement of gas chromatography, numerous analytical methods have been developed for determining AA concentration in various food samples. Table 1 provides an overview of selected methodologies. Gas chromatography, in particular, is a crucial tool for ensuring the quality of food products (Pastor et al., 2019). Chromatographic analysis, known for its sensitivity, often requires additional sample preparation steps such as derivatization which promote volatility and thermal stability of compounds. Initially, derivatization procedures involved the use of toxic bromine reagents. However, a recent study employing gas chromatography-electron capture detection (GC-ECD) introduced a more environmentally friendly derivatization procedure (Saraji & Javadian, 2019). This method utilized a mixture of hydrobromic acid and ammonium persulfate to perform the derivatization reaction at a relatively low temperature of 45 °C. Additionally, GC combined with a nitrogen phosphorus detector and solid phase extraction was found to quantify acrylamide with a detection limit of 0.012 ppm within a concentration range of 0.1–5 ppm (Alnedhary et al., 2023).

**Table 1:** Comparison of GC experimental procedures and parameters.

Detection	Mobil phase; flow rate	Stationary phase	Injector temperature	Time (total experiment)	LOD	LOQ	Ref.
FID	Hydrogen (H_2_)	DB-FFAP column	NA	NA	27.4 μg/kg	91.5 μg/kg	(Passos et al., 2021)
	Helium (He); 2.9 mL/min	CP-Sil 8CB column	280 °C	NA	0.2–2 μg/kg	0.6–6.7 μg/kg	(Seilani et al., 2021)
EGC	N_2_	DB-1701 GC column	240 °C	10 min	0.60 μg/L	2.0 μg/L	(Saraji & Javadian, 2019)
MS	He; 1.2 mL/min	HP-INNOWax column	230 °C	20 min	3 μg/kg	10 μg/kg	(Roszko et al., 2020)
	He; 1.7 mL/min	DB-FFAP column	NA	NA	0.11 μg/vial	0.37 μg/vial	(Passos et al., 2021)
	He	Agilent VF-WAXms capillary column	200 °C	20 min	4 μg/kg	11 μg/kg	(Brenes-Álvarez et al., 2023)
	He; 1 mL/min	CD-5MS capillary column	220 °C	14 min	3 mg/kg	10 mg/kg	(Kang et al., 2020)
	He; 0.8 mL/min	HP-5 ms capillary column	280 °C	NA	20 ng/g	66 ng/g	(Aghvami et al., 2023)
	He; 0.8 mL/min	HP-5 MS capillary column	290 °C	12 min	0.6 μg/kg	2 μg/kg	(Nematollahi et al., 2020)
	He; 0.8 mL/min	HP-5 MS capillary column	290 °C	12 min	0.59 ng/g	1.99 ng/g	(Kamankesh et al., 2021)
MS/MS	He; 1 mL/min	Trace GOLD™ TG-WAX GC Column	240 °C	21 min	6–12 μg/kg	28–34 μg/kg	(Hasan et al., 2022)
	He; 1.6 mL/min	TraceGOLD™ TG-WaxMS capillary column	220 °C	13 min	1.23 μg/kg	3.7 μg/kg	(Negoiță et al., 2020)

Recent studies have indicated that AA can be detected even using the cost-effective gas chromatographs coupled with flame ionization detectors (GC-FID). In particular, green microextraction methods, like dispersive liquid-liquid microextraction (DLLME), have been proposed for the extraction of AA from water samples. When combined with GC-FID, this method provides a low-cost analytical option with acceptable detection limits (Sayah & Kiarostami, 2019). Additionally, recent studies have successfully quantified underivatized acrylamide in biscuit samples following headspace-solid phase microextraction, with optimal results achieved using CW/DVB and CAR/PDMS bipolar fibers (Passos et al., 2021). Furthermore, GC-FID has been utilized to assess the impact of cooking methods and durations on acrylamide concentrations. Results from QuEChERS extraction revealed that traditional frying methods and prolonged cooking times significantly elevated AA levels. Specifically, shrimp nuggets fried using the traditional method (220 °C for 6 min) exhibited the highest acrylamide concentration (27 ng/g), while chicken nuggets fried using the industrial method (180 °C for 3 min) contained the lowest levels (7.3 ng/g) (Seilani et al., 2021).

The solubility of acrylamide complicates its extraction from food, as residual water often remains in the extract. However, for gas chromatography–mass spectrometry (GC–MS) preparation, the residual water must be removed to prevent damage to the column from thermal expansion. The standard method involves salting out with sodium chloride and magnesium sulfate, enabling acrylamide to partition into the acetonitrile layer due to a density gradient (Cantrell & McDougal, 2021).

Compared to the other GC detection methods, MS is more frequently used in studies analyzing acrylamide in food products due to its great selectivity and sensitivity in detecting small molecule. However, pretreatment steps such as derivatization, necessary to avoid interference from co-extractives and increase the volatility of acrylamide, can become burdensome for routine analyses. For instance, detecting acrylamide in black ripe olives requires a derivatization step to convert it to 2-bromopropenamide before GC–MS analysis (Brenes-Álvarez et al., 2023). Furthermore, the use of potassium bromide and potassium bromate under acidic conditions can generate 2,3-dibromopropanamide as AA derivative. Combined with automatic accelerated solvent extraction in ethyl acetate and GC–MS the method is able to detect AA with a limit of detection of 3 μg/kg (Kang et al., 2020). Xanthydrol can also be considered as a derivatization reagent for qualitative analysis of AA. For example, a recent study that evaluated the effect of flavors on AA levels on cake showed that, after derivatization and GC–MS analysis, the highest levels of AA were related to the cinnamon cakes (212.28 ng/g) (Aghvami et al., 2023). Similarly, *N*,*O*-bis(trimethylsilyl)trifluoroacetamide was found to increase sensitivity of GC–MS method while studying AA levels in coffee and coffee substitutes (Surma et al., 2017).

In addition to derivatization, GC requires purification steps for substances that could be co-eluted from the crude matrices. In the case of aqueous extractions, it is necessary to remove residual sugars and asparagine that could otherwise generate distorted results in a heated GC injector. In this sense, a procedure based on solid phase extraction with ion exchange was used to analyze AA concentrations in different soft bread samples. AA levels varied greatly from sample to sample (in the range of 3.6–163 μg/kg), suggesting that both food composition and manufacturing processes play a crucial role in AA generation (Roszko et al., 2020). Recently, a dispersive liquid–liquid microextraction method combined with solidification of aqueous phase prior to GC–MS detection generated a limit of detection of 0.2 μg/L for AA in vegetable and fruit chips (Khongsiri et al., 2024). Additionally, the pH of the sample solution is crucial in microextraction procedures, as it influences the transfer of analyte molecules into the organic phase. A recent study using roasted nuts and seeds as food samples found that a pH of 7 is the most suitable for dispersive liquid-liquid microextraction (Nematollahi et al., 2020).

Snack samples, known as carbohydrate- and protein-rich food, have high potential to contain acrylamide. In a recent study, that applied microextraction technique coupled with gas chromatography–mass spectrometry, maximum amounts of 1183 ng/g acrylamide were detected for potato crisps. Various parameters were optimized using response surface methodology based on central composite design in order to obtain a limit of detection and quantitation of 0.59 ng/g and 1.99 ng/g, respectively (Kamankesh et al., 2021). Furthermore, ultrasound extraction has proven effective in isolating acrylamide from heat-treated, carbohydrate-rich foods. After hexane defatting, bromination, and GC–MS/MS detection, the results indicated that raw materials significantly impact acrylamide levels, as different ingredients contain varying amounts of free asparagine and reducing sugars (Hasan et al., 2022).

The desire to reduce sample preparation steps lead to the development of solid-phase extraction (SPE) procedures. A recent study highlighted the optimization of clean-up methods, achieving maximum efficiency using SPE cartridges (Negoiță et al., 2020). It was found that extracting and cleaning up AA from chips and French fries samples was most efficient when performed with water at room temperature and 10 mL of hexane, omitting the use of Carrez solutions. The clean-up process utilized two SPE cartridges with an optimal elution solvent volume of 5 mL, resulting in highly efficient analyte extraction. Alternatively, cost-effective liquid-liquid extraction can be used as a simplified method for evaluating AA levels. This approach has already demonstrated its efficiency in analyzing instant coffee products by GC–MS (Karami et al., 2022).

### 3.2 Liquid chromatographic methods

While advanced techniques like liquid chromatography coupled to mass spectrometry (LC–MS, GC–MS, LC–MS/MS) or ultra-performance liquid chromatography/tandem mass spectrometry (UPLC–MS/MS) are available for AA analysis in various food products, as observed in Table 2, their high cost and limited accessibility in developing countries lead many researchers to favor the more economical reversed-phase chromatography (Oroian et al., 2015). Specifically, in the context of a diode array detector (DAD), the analytical method recommends monitoring AA at 210 nm and 284 nm, which corresponds to its maximum absorption wavelengths (Shi et al., 2019). An alternative method, recently optimized, detects acrylamide solely at the wavelength of 210 nm, exhibiting a limit of detection (LOD) and limit of quantitation (LOQ) of 3.733 and 11.045 ng/μL, respectively (Verma & Yadav, 2022). In the assessment of acrylamide levels in roasted date seed samples, an advanced QuEChERS method, coupled with the HPLC–PDA technique, detected acrylamide traces at a wavelength of 202 nm, establishing a correlation with the browning index (Khaloo Kermani et al., 2023).

**Table 2:** Comparison of LC experimental procedures and parameters.

Detection	Mobil phase; flow rate	Stationary phase	Temperature column	Time (total experiment)	LOD	LOQ	Ref.
DAD	5 % acetonitrile (ACN) in water (H_2_0); 0.6 mL/min	C18 ODS Hypersil column	40 °C	15 min	6.90 μg/L	20.90 μg/L	(Shi et al., 2019)
	90:10 H_2_0:ACN; 1 mL/min	Zorbax column	40 °C	15 min	3.733 ng/μL	11.045 ng/μL	(Verma & Yadav, 2022)
	99 % H_2_0 and 0.1 % acetic acid solution; 0.50 mL/min	Agilent ZORBAX Eclipse plus column	30 °C	15 min	5 μg/L	20.40 μg/kg	(Endeshaw & Belay, 2020)
PDA	3:97 ACN:H_2_0; 0.7 mL/min	Eurospher 100–5 C18 column	NA	40 min	7.73 μg/L	25.76 μg/L	(Khaloo Kermani et al., 2023)
MS	0.06 % formic acid (FA) and methanol (MeOH); 0.4 mL/min	Spherisorb ODS-2 column	35 °C	44 min	4 μg/kg	11 μg/kg	(Brenes-Álvarez et al., 2023)
	0.1 % FA and MeOH: ACN (50/50, *v*/v) with 0.1 % FA; 0.4 mL/min	Agilent C18 Eclipse Plus column	NA	40 min	1.8 μg/kg	6 μg/kg	(Crawford et al., 2019)
MS/MS MS/MS	0.1 % FA and MeOH with 0.1 % FA	Synergi Hydro RP column	NA	25 min	26.7 ppb	NA	(Ledbetter et al., 2020)
	0.1 % of FA and MeOH; 0.4 mL/min	ACE Excel 3 SuperC18 column	30 °C	20 min	5 μg/kg	20 μg/kg	(Prata et al., 2023)
	0.1 % FA and MeOH; 0.5 mL/min	Zorbax Eclipse XDB-C18 column	NA	27 min	NA	NA	(Brenes-Álvarez et al., 2023)
	ACN and 0.01 mM acetic acid, 0.2 % FA; 0.3 mL/min	Zorbax Extend-C18 column	25 °C	20 min	0.62 ng/g	1.89 ng/g	(Jozinović et al., 2019)
	ACN with 0.05 % FA and 0.05 % FA; 0.3 mL/min	Agilent poroshell C-18 column	NA	10 min	0.7 μg/kg	2.0 μg/kg	(Kumari et al., 2023)
	H_2_O and MeOH; 0.6 mL/min	Shodex RSpak DE-413 column	40 °C	10 min	NA	0.2 μg/kg	(Bebius et al., 2024)
	H_2_O; 0.15 mL/min	Hypercarb column	NA	NA	1 μg/kg	25 μg/kg	(Nur Hidayah et al., 2024)
	0.1 % FA in H_2_O and 0.1 % FA in MeOH; 0.25 mL/min	Zorbax XDBC18 column	30 °C	NA	NA	NA	(Lodolini et al., 2019)
	0.5 % MeOH and 0.1 % acetic acid; 0.3 mL/min	Kinetex polar C18 column	26 °C	NA	0.2 μg/mL	0.67 μg/mL	(S. Choi et al., 2024)
HRMS	2 % MeOH and 98 % MeOH containing 5 mM ammonium formate and 0.1 % FA; 300 μL/min	C18 UHPLC column	30 °C	15 min	NA	200 mg/kg	(España Amórtegui et al., 2024)

The main detectors used in HPLC (e.g. UVD, FLD, and DAD) have the drawback of low sensitivity and in some cases cannot adequately identify the interfering components that are co-extracted with target analytes (Jozinović et al., 2019). However, mass spectrometry can significantly improve sensitivity. HPLC-MS and HPLC-MS/MS are the most often used methods as they enable favorable separation and unambiguous determination of a great number of newformed contaminants in the food matrix without prior derivatization. In mass spectrometric analysis, acrylamide can be quantified using the *m*/*z* 72 → m/z 54.8 transition (Brenes-Álvarez et al., 2023; Crawford et al., 2019). However, HPLC-MS/MS is used more widely than HPLC-MS due to its higher selectivity, sensitivity, and anti-interference capability.

In addition to AA, the Maillard reaction can generate 5-hydroxymethylfurfural (HMF) and heterocyclic aromatic amines (HAAs) like 2-amino-1-methyl-6-phenylimidazo [4,5-b] pyridine (PhIP). Thus, various methods for the simultaneous detection of harmful compounds produced during food thermal processing were developed. The UPLC-MS/MS technique allows for the simultaneous detection of these compounds, overcoming the low sensitivity limitations observed in classical detectors. For example, by using an isocratic gradient with a duration of only 20 min, acrylamide and HMF were determined simultaneous in corn snack products (Jozinović et al., 2019). Other studies focused on optimizing the pretreatment steps, indispensable before instrumental analysis. Thus, novel cysteine (Cys)-functionalized magnetic covalent organic frameworks were synthesized and used as adsorbents for magnetic solid-phase extraction, generating a reliable method for the simultaneous detection of AA and 5 HAAs in thermally processed foods by HPLC-MS/MS (Feng et al., 2023).

The challenge of devising a sensitive, precise, and robust method for acrylamide detection and quantification has persisted among researchers. A recent study has successfully introduced and validated an LC-MS/MS approach for assessing acrylamide content in potato-based snacks, achieving a remarkable detection capability as low as 0.7 μg/kg, and a reporting limit of 2.0 μg/kg (Kumari et al., 2023). The minimization of interference was achieved through the implementation of effective sample cleaning procedures. Moreover, a mass spectrometry technique utilizing selective reaction monitoring was found to enabled clear identification of the target compound, even at low limits of detection (LODs) and quantitation (LOQs) of 5 and 20 μg/kg, respectively (Prata et al., 2023). In addition, the application of LC-MS/MS for acrylamide quantification has proven instrumental in identifying key factors that contribute to acrylamide formation during potato crisp production (Ledbetter et al., 2020).

The assessment of acrylamide levels in coffee via LC-MS/MS is vulnerable to interference issues, potentially resulting in overestimation of up to 40 % due to coelution with unidentified background ions (Desmarchelier et al., 2020). To address this concern, adjusting the mobile phase pH proved effective in minimizing interference and ensuring accurate acrylamide quantification. Moreover, compounds closely resembling acrylamide were observed to undergo fragmentation during analysis, underscoring the critical role of chromatographic conditions in achieving reliable quantification (Desmarchelier et al., 2020). Various methods highlighted overestimation challenges in cocoa samples and emphasized the influence of chromatographic conditions on quantification across diverse food products. Subsequent evaluation of interference in coffee, cereal-based products, and baby foods aimed to rectify cases of misquantification and validate a robust method for interference-free acrylamide analysis, demonstrating exceptional performance across multiple food matrices (Desmarchelier et al., 2022). Furthermore, a recent quantitative LC-MS/MS method for acrylamide determination in food successfully analyzed up to 16 individual laboratory samples across nine matrix categories, meeting all specified performance criteria for limits of quantification, recovery, precision, and accuracy (Bebius et al., 2024). Another strategy addressed to reduce potential interfering substances involved forming and quantifying 2,3-dibromoacrylamide, achieved by bromo-derivatizing of acrylamide under acidic conditions (Wang et al., 2022). Moreover, the use of 100 % water mobile phase in chromatographic separation was found to prevent the co-elution of *N*-acetyl-β-alanine and acrylamide in a broad range of food matrices (Desmarchelier et al., 2022).

## 4 Strategies for mitigating acrylamide formation in food products

According to the European Food Safety Authority (EFSA), the tolerable daily dose of acrylamide is 170 μg/kg of body weight for tumor risk and 430 μg/kg for neurological changes. The rising consumption of fried and baked foods has elevated concerns regarding acrylamide mitigation within the food processing industry. The formation of acrylamide varies depending on the type of food, being mainly influenced by the Maillard reaction, which occurs at temperatures above 120 °C (Sarion et al., 2021). This mechanism is predominant in carbohydrate-rich foods such as potato products, grain products, or coffee subjected to heat treatment. For example, one study showed that AA level in a bread sample baked at 220 °C for 60 min reached 85.05 ± 4.13 μg/kg, significantly higher than the 39.32 ± 1.90 μg/kg obtained at 180 °C with a baking time of 40 min (Jensen et al., 2024).

Interestingly, increasing the intensity of the heat treatment can also contribute to the reduction of acrylamide levels through processes such as thermal degradation and evaporation. A relevant example is coffee roasting, where it was observed that more intensely roasted beans (260 °C for 15 min) contain lower levels of acrylamide compared to those that are lightly roasted (240 °C for 5 min) (Schouten et al., 2020). However, excessive baking, although it can reduce certain harmful compounds, negatively affects the quality and edibility of the final product, which makes this method not considered an effective strategy for acrylamide reduction.

**Fig. 2:**
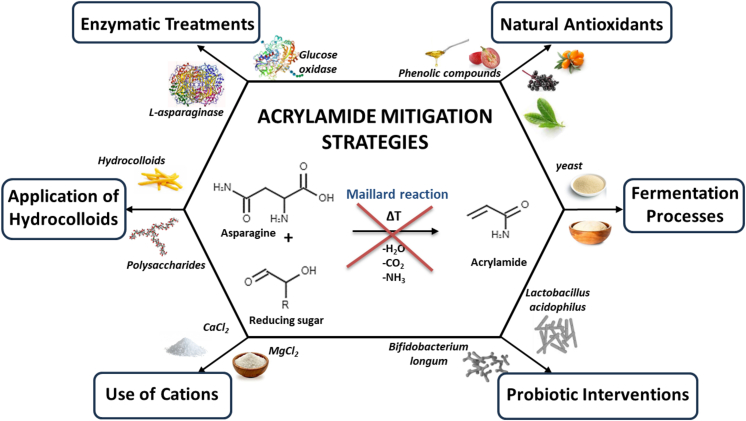
Innovative strategies used to lower acrylamide levels in foods.

As previously exemplified, various strategies can be applied to reduce acrylamide levels, such as adjusting heating time and temperature, controlling levels of reducing sugars and amino acids, and adjusting pH. However, these treatments can also negatively impact the sensory qualities of food, particularly taste. To address this, multiple pre-treatments have been identified as effective in reducing AA levels (Kathuria et al., 2023). Examples of technological and biochemical approaches for reducing acrylamide in foods are summarized in Table 3. These include blanching, soaking, using salt solutions, monitoring storage temperatures of raw materials, adding competing amino acids, antioxidants, enzymes and organic acids for pH reduction (See Fig. 2).

**Table 3:** Examples of technological and biochemical measures for reducing acrylamide in foods.

Strategy	Mechanism	Application Examples	Remarks	References
Lowering frying/baking temperature	Reduces Maillard reaction kinetics by lowering thermal input	Bread at 180 °C/40 min: 39.32 μg/kg AA vs. 220 °C/60 min: 85.05 μg/kg	Effective AA reduction; may compromise sensory qualities	(Jensen, Hakme, & Feyissa, 2024)
Enhanced thermal processing	Accelerates acrylamide degradation and evaporation	Coffee roasted at 260 °C/15 min reduced AA versus 240 °C/5 min	Risk of flavor degradation and overprocessing	(Schouten, Tappi, & Romani, 2020)
Enzymatic reduction of L-asparagine by natural L-asparaginases	Hydrolysis of free L-asparagine by native enzymes	Potato slices 37–60 °C/10–30 min reduced AA 20–65 %; fries preheated 80 °C/10 min reduced AA >80 %	Enzyme stability and pH sensitivity limit applications; process conditions critical	(Chi et al., 2021; Onishi et al., 2015; Saeed et al., 2018; Sajed et al., 2022)
Recombinant or engineered L-asparaginase	Engineered enzymes with enhanced stability and specificity	Mutants reduced AA up to 98 % in potato chips, bread	Improved industrial applicability; sensory impact and scale-up require further study	(Akwagiobe et al., 2023; Chi, Zhu, et al., 2023; Jiao et al., 2020; Yuan et al., 2022)
Substrate manipulation: glucose removal	Enzymatic glucose degradation to limit acrylamide formation	Glucose degradation lowered AA from 1346 to 424 mg/kg	Supports combined substrate and enzymatic treatments	(El-Sayed, El-Maaty, & Abdelhady, 2023; Hamzalıoğlu & Gökmen, 2024; Sun et al., 2020)
Combined enzymatic treatment	Dual enzymatic degradation of asparagine and glucose	Starchy foods at 37 °C/30 min reduced AA by ∼70 %	Synergistic AA reduction; broad industrial potential	(El-Sayed, Abdelhady, et al., 2023)
Microbial degradation of acrylamide	Acrylamidase cleaves AA to ammonia and acrylic acid	Complete AA degradation in coffee and wastewater within 60 min	Product safety evaluation needed; industrial validation required	(Bedade, Muley, & Singhal, 2019; Bedade & Singhal, 2017; Bedade, Sutar, & Singhal, 2019; Kulkarni et al., 2020)
Natural antioxidants (green tea, grape seed)	Scavenge radicals and react with precursors to inhibit AA formation	Green tea polyphenols reduced AA by up to 64 %	Effects depend on antioxidant type, dose, and food matrix	(Torres et al., 2019; Ma et al., 2020)
Olive-derived polyphenols	Phenolics inhibit acrylamide and acrolein formation	Tyrosyl acetate reduced AA by up to 90 %; olive leaf extracts reduced AA by 54 %	Highly effective phenolics; suitable for oil-rich foods	(Pantalone et al., 2021(Pantalone et al., 2021; Mechi, Fernández, Baccouri, Abaza, & Martín-Vertedor, 2022); Martín-Vertedor et al., 2020)
Use of phenolic-rich plant extracts or powders	Phenolics interrupt AA formation while preserving sensory quality	Onion peel powder, buckwheat extract, wild berries used in baked goods	Effective AA reduction without sensory compromise	(Borczak et al., 2022; Jing et al., 2019; Yeom & Hwang, 2020)
Yeast and sourdough fermentation	Fermentation reduces free asparagine	Baker's yeast reduced AA ∼80 % in fried potato chips	Practical and effective for starchy foods	(El-Sayed, El-Maaty, & Abdelhady, 2023; Lopez-Moreno et al., 2023)
Use of specific microbial strains	LAB and yeast produce acids lowering pH and AA formation	Selected LAB strains reduced AA up to 79.6 %	Strain selection and combination key for effectiveness	(Ameur et al., 2024; Nasiri Esfahani et al., 2017; Zhou et al., 2022)
Use of pseudo-cereal doughs and sprouted grains	Fermentations reduce AA and precursor compounds	Lupine/flaxseed biscuits reduced AA by 78–85 %	Improves safety and nutrition; monitoring HMF levels necessary	(Bartkiene et al., 2016; Yıltırak et al., 2021)
Probiotics (*L. acidophilus*, *L. plantarum*)	Amidase activity degrades AA during digestion or processing	Probiotic strains reduced AA by up to 52 %	Efficacy influenced by strain, pH, and nutrient conditions	(Choi et al., 2022; Choi et al., 2023; Petka et al., 2021; Pop et al., 2022)
Divalent cations (Ca^2+^, Mg^2+^)	Modify Maillard pathway to favor caramelization over AA	CaCl₂ in masa; MgCl₂ in frying and cereals	MgCl₂ more effective; may affect flavor and increase HMF	(Arámbula-Villa et al., 2018; Kukurová & Ciesarová, 2024
Hydrocolloid coatings	Retain moisture and suppress Maillard reaction	Coated fries, nuggets, biscuits reduced AA by 35–70 %	Often combined with plant extracts for enhanced effect	Huang et al., 2022; Zokaei et al., 2020; Al-Asmar et al., 2018; Jiang et al., 2021; Mousa, 2019; Champrasert et al., 2021)
Enzyme-loaded biopolymer hydrocolloids	Protective carriers shield L-asparaginase and act as heat barriers	Zein-pectin films reduced AA by 70 % in baked goods	Synergistic effect improving enzyme stability and activity	(Bisht et al., 2024; Champrasert et al., 2022)

### 4.1 Enzymatic treatments

Free asparagine is crucial for acrylamide formation, leading recent studies to explore enzymatic treatment with L-asparaginase to reduce L-Asn levels. For example, treatment of sliced potatoes with *Bacillus subtilis* L-asparaginase II (incubated for 10 min at 60 °C) demonstrated a significant reduction in acrylamide content in fried potato chips (fried at 170 °C for 90 s), reducing it to less than 20 % compared to that in untreated chips (Onishi et al., 2015). However, the study does not investigate the reproducibility of enzyme performance across different food matrices which restrict the generalizability of the findings. Despite its recognized efficacy in acrylamide prevention and leukemia treatment, the industrial use of L-asparaginase is hindered by its narrow pH stability and low thermostability. However, a novel L-asparaginase from *Mycobacterium gordonae* (GmASNase), cloned and expressed in *Escherichia coli* BL21 (DE3), has shown high specific activity and excellent stability across a pH range of 5.0–11.0, with optimal activity below 40 °C (Saeed et al., 2018). Application of GmASNase to potato chips prior to frying (180 °C for 5 min) resulted in a significant 65.09 % reduction in acrylamide content compared to untreated samples, presenting a more reliable solution for acrylamide mitigation (Chi et al., 2021). This result can be attributed, in part, to the extended incubation time of 30 min and the temperature favorable for enzymatic activity of 37 °C. However, further research is required to evaluate enzyme stability under continuous industrial-scale processing. Similarly, Tk2246, a plant-derived L-asparaginase from *Thermococcus kodakarensis*, expressed in *Escherichia coli*, demonstrated remarkable efficiency, reducing acrylamide formation by over 80 % in various foods including French fries, chapati, and yeast-leavened bread. For French fries, the treatment included a pre-incubation with Tk2246 at 80 °C for 10 min, while for the other products, the enzyme was used as a direct additive in the recipe, thus simplifying the preparation process. In addition, Tk2246 exhibited anti-staling properties without compromising sensory attributes (Sajed et al., 2022). However, the impact of high incubation temperature (80 °C) on enzyme structure and shelf life is not addressed, raising questions regarding long-term storage and formulation stability.

Due to its low substrate affinity and selectivity, recent research (Jiao et al., 2020) has focused on the enhancement of L-asparaginase. Thus, cloning and expression of the type I L-asparaginase gene from *Acinetobacter soli* in *Escherichia coli* generated a recombinant enzyme with high specificity. It showed no activity towards l-glutamine or D-asparagine, significantly increasing its potential for industrial applications. However, the enzyme's poor thermostability limited its efficiency in degrading acrylamide in potato chips fried at 170 °C for 5 min, despite preincubation at only 37 °C for 30 min.

In efforts to enhance L-asparaginase performance, a targeted engineering approach was employed to improve thermostability by identifying and mutating critical residues, namely Gly97, Asn159, and Glu249. This led to the creation of a combinatorial triple mutant G97T/N159Y/E249Q, which demonstrated significantly enhanced thermostability due to a more rigid enzyme structure, with half-life values of 61.65 ± 8.69 min at 50 °C and 5.12 ± 1.66 min at 55 °C (Chi, Zhu, et al., 2023). Similarly, an *Escherichia coli* L-asparaginase stable variant, D60W/L211R/L310R, was developed using molecular dynamics simulation, saturation mutation, and combinatorial mutation techniques. The application of this stable mutant at concentrations of 60 U/mL and 100 U/g flour under uncontrolled temperature and pH conditions resulted in a reduction of acrylamide content by 66.9 % in potato chips and 51.7 % in bread (Yuan et al., 2022). While promising, both studies lack detailed sensory analysis post-treatment, which is critical for consumer acceptability. Additionally, consensus design was employed to improve the thermal stability of *Corynebacterium glutamicum* L-asparaginase, leading to the double-mutant enzyme L42T/S213N, which showed a 3.1-fold increase in half-life at 40 °C without compromising activity (Chi, Jiang, et al., 2023). Homology modeling identified six amino acid mutations (V26A, E30G, K122N, D181G, V245G, G276D) associated with heightened catalytic activity (Lu et al., 2019), with DD-12G showing notable improvements in pH and temperature tolerance. Structural analysis indicated reduced hydrogen bonds and increased flexibility near the active site, enhancing catalytic efficiency. Additionally, advancements in strain improvement through atmospheric pressure and room temperature mutagenesis, combined with batch mode bioreactor fermentation, yielded the Val-Asp-S-180-L mutant with a 2.5-fold increase in L-asparaginase activity (Akwagiobe et al., 2023). Despite its significance, this finding is limited to a single food type and requires broader validation to ensure generalizability. Operating optimally at acidic pH and 65 °C, this mutant achieved a substantial 98.18 % reduction in acrylamide levels in sweet potato chips. Moreover, in silico methodologies can complement experimental approaches by providing insights into enzyme behavior at the molecular level, thereby facilitating the design and optimization of enzymes, as demonstrated in studies showcasing the enhanced protein and thermal stability of a 10-mutant variant of L-asparaginase (Sundaram et al., 2024). However, experimental confirmation of in silico predictions remains limited, which reduces confidence in their immediate applicability.

The mechanism of acrylamide formation may likely involve the pyrolysis of asparagine in the presence of glucose (Hamzalıoğlu & Gökmen, 2024). The observed positive correlation between glucose concentration and acrylamide levels (r^2^ = 0.61) suggests that reducing glucose levels could lower acrylamide formation (Sun et al., 2020). This hypothesis was tested by researchers, with some studies confirming a reduction in acrylamide levels from 1346.0 mg/kg in the control sample to 424.3 mg/kg following the degradation of glucose using enzymes such as glucose oxidase (El-Sayed, El-Maaty, & Abdelhady, 2023).

Enzymatic treatment has shown significant efficacy, even when used in combination. In a recent study employing a 1:1 mixture of L-asparaginase and glucose oxidase, acrylamide levels were reduced from 865 mg/kg in the control sample to 260 mg/kg in the mixed enzyme-treated sample and 215 mg/kg in the green tea powder-treated sample following incubation at 37 °C for 30 min (El-Sayed, Abdelhady, et al., 2023). L-asparaginase alone reduced acrylamide by 67.63 %, while the mixed enzyme blend achieved a 69.94 % reduction. The study concluded that these treatments can substantially lower acrylamide levels in starchy foods without compromising quality, providing a method to protect school-age children and youth from 70 to 80 % of their daily acrylamide intake.

Despite acrylamide's general toxicity in its monomeric form, specific microorganisms possess the capability to utilize it as an energy substrate. This metabolic process involves the enzymatic breakdown of acrylamide into ammonia and acrylic acid, facilitated by acrylamidase derived from the Gram-positive bacterium *Arthrobacter* sp. DBV1 (Bedade & Singhal, 2017). In another study, Bedade, Muley, and Singhal (2019) explored the immobilization of bacterial acrylamidase from *Cupriavidus oxalaticus* ICTDB921 on chitosan-coated alginate beads to reduce AA content (Bedade, Sutar, & Singhal, 2019). However, the absence of safety data regarding degradation by-products, particularly acrylic acid, and the lack of toxicological evaluation of treated samples are absent, limiting regulatory approval.

The immobilized acrylamidase demonstrated optimal pH and temperature conditions (8.5/65 °C), enhanced pH and thermal stability, and maintained 80 % activity over four cycles. Applied to instant coffee, this treatment resulted in complete acrylamide degradation within 60 min from initial concentrations of 100–500 μg/L. Additionally, the use of acrylamidase from *Cupriavidus oxalaticus* was extended to industrial wastewater, where the enzyme immobilized on magnetic nanoparticles achieved complete degradation of 2100 mg/L acrylamide (Bedade, Muley, & Singhal, 2019). Another approach involved the use of stabilized cross-linked enzyme aggregates of acrylamidase for treating industrial wastewater, resulting in the complete degradation of 1.75 g/L acrylamide within 60 min under optimal conditions (Kulkarni et al., 2020). While these results highlight the high catalytic efficiency of immobilized acrylamidase systems under controlled conditions, their scalability, long-term use and performance in complex real food matrices remain largely untested.

### 4.2 Natural antioxidants

Bioactive phytochemicals, including phenolic acids, flavonoids, anthocyanins, and carotenoids, are highly regarded for their robust antioxidant properties. The use of plant extracts as a source of bioactive compounds is an increasingly promising strategy for enhancing the quality and functional attributes of foods, while also contributing to increased health benefits. Due to their natural origin, these bioactive compounds are considered ideal alternatives to synthetic antioxidants, which are often perceived as less safe.

The relationship between antioxidants and acrylamide formation is a highly controversial topic in the literature, as their effects vary depending on the type, concentration and specific conditions of the food process (Zhang and Jin, 2024). Although it has been shown that some antioxidants can inhibit acrylamide formation by reacting with their precursors, others can enhance acrylamide synthesis by acting at different stages of the chemical reactions involved, such as the Maillard reaction or the oxidation of acrolein (Ghanati et al., 2024). This variability can be attributed both to the distinct chemical characteristics of the antioxidants and to the diverse experimental conditions of the studies, including the preparation methods and concentrations applied. For instance, green tea polyphenols have been shown to reduce acrylamide formation in baked starchy products by approximately 48 %, with this reduction further enhanced to 64 % when combined with inulin, indicating the potential of functional ingredients in improving the health profile of starchy bakery items (Torres et al., 2019). On the other hand, the use of grape seed extract highlights a nonlinear relationship between the concentration of antioxidants and their inhibitory effect (Ma et al., 2020). The observation that the lower dose (1.25 g/kg) resulted in a greater reduction in acrylamide (58.23 %) than the higher dose (2.5 g/kg - 33.32 %) is intriguing, but also problematic. This inverse relationship could be influenced by factors such as the optimal concentration of quercetin required to intercept acrylamide precursors or the possible auto-oxidation of polyphenols at higher concentrations.

It is known that the use of vegetable oils in food processing, especially at high temperatures (150–200 °C), contributes to the development of desired sensory properties, but also generates degradation processes that can produce toxic compounds, such as acrolein (Zhang and Jin, 2024). However, beneficial phenolic compounds from olive oil by-products, such as tyrosyl acetate (TyAc) and hydroxytyrosyl acetate, have been shown to be effective in reducing acrylamide levels, with TyAc achieving a reduction of up to 90 % (Pantalone et al., 2021). Other substances, such as tyrosol, hydroxytyrosol, caffeic acid and oleuropein, had more moderate effects, while olive leaf extracts, in combination with hydroxytyrosol, reduced acrylamide levels by up to 54 % (Mechi et al., 2022; Martín-Vertedor et al., 2021).

In the context of bakery products, recent studies have shown interesting results from the addition of phenolic-rich ingredients. For example, the incorporation of 6 % onion peel powder in cookies (Yeom & Hwang, 2020) or the addition of 200 μl (1 mg/mL) buckwheat extracts in bread (Jing et al., 2019) not only reduced acrylamide content, but also preserved the sensory and textural attributes of the products. These effects are likely attributed to the antioxidant activity of phenolic compounds, which may interact with acrylamide precursors or intermediate radicals during the Maillard reaction, thus interrupting acrylamide formation. However, the exact mechanism by which these phenolics influence acrylamide pathways remains unclear, as most studies report empirical results. Similarly, the addition of 5 g phenolic-rich wild berries such as wild rose, elderberry, sea buckthorn, rowan, chokeberry and hawthorn to wheat flour cookies resulted in good antioxidant activity and a significant reduction in acrylamide content, compared to control cookies (Borczak et al., 2022). On the other hand, another study showed that sweet and savoury biscuits enriched with 10 % nuts, dried fruits, dried seeds or black olives, ingredients that also contain phenolic compounds, had significantly higher concentrations of acrylamide than the control variant, due to the presence of its precursors or, sometimes, of already formed acrylamide, due to processing processes (Schouten et al., 2024). This suggests the need to monitor not only the presence of acrylamide in some, but also how the phenolic compounds in these ingredients interact with other components in recipes rich in the ingredients at different processing stages.

### 4.3 Fermentation processes

Fermentation is a promising method for mitigating acrylamide formation in food products, primarily through the absorption and reduction of asparagine, a key precursor in the acrylamide formation process. In recent studies, various fermentation techniques have demonstrated significant reductions in acrylamide content across different food matrices.

Baker's yeast has been particularly effective in reducing acrylamide levels. One study reported an 80 % reduction in acrylamide production in potato chips treated with baker's yeast following frying at 180 ± 5 °C for 10 min (El-Sayed, El-Maaty, & Abdelhady, 2023). This highlights the potential of yeast fermentation as a practical approach for decreasing acrylamide in heat-treated foods.

Sourdough and extended fermentation are potential strategies for reducing acrylamide formation in bread. Recent studies have shown that extending the fermentation time to 360 min (with humidity of 0 ± 2 % and temperature of 30 ± 1 °C) significantly reduced acrylamide levels in bread with 0 % and 20 % dough (Lopez-Moreno et al., 2023). However, for bread containing 50 % dough, acrylamide levels varied depending on the stage of fermentation. This variability may be associated with the amylolytic and proteolytic activities of lactic acid bacteria, which had a major impact on acrylamide formation.

The incorporation of specific strains in sourdough fermentation has been demonstrated to significantly reduce acrylamide content in breads. Utilizing *Lactobacilli* and *Saccharomyces cerevisiae* in the fermentation process effectively diminishes acrylamide levels (Zhou et al., 2022). This reduction was primarily achieved through the lowering of pH, as these microorganisms produce organic acids via their metabolic activities, thereby mitigating acrylamide formation. Similarly, tailored sourdough fermentations offer an effective biotechnological strategy for reducing acrylamide levels in food products. A study investigating various strains of lactic acid bacteria (LAB) in the preparation of rye sourdough revealed a significant decrease in acrylamide concentrations in rye crispbread, with reductions of up to 79.6 % compared to the baker's yeast control (Ameur et al., 2024).

Alternative strategies employing combinations of sourdough and yeast demonstrated that acrylamide levels in bread fermented with sourdough and yeast were consistently lower than those in bread fermented with yeast alone. This reduction in acrylamide content was particularly pronounced when appropriate *Lactobacillus* strains were utilized in the sourdough fermentation process (Nasiri Esfahani et al., 2017).

The use of pseudo-cereals in wheat products aims to enhance the nutritional value of wheat flour, although plant additives rich in proteins can increase acrylamide content in baked goods. A study focused on reducing acrylamide in wheat flour biscuits supplemented with lupine and defatted flaxseed flour through solid-state and submerged fermentations using *Lactobacillus sakei*, *Pediococcus pentosaceus*, and *Pediococcus acidilactici* found that post-fermentation asparagine decreased by an average of 67.6 % and 80.6 % (Bartkiene et al., 2016). The most effective acrylamide reduction in biscuits (78 % and 85 %) was achieved using *P. acidilactici* for flaxseed and lupine, suggesting that these compounds could be used to produce safer, high-nutritional-value biscuits.

The use of sprouted grains in thermally processed foods is gaining popularity due to the in sugar and amino acid composition, which affect Maillard reaction precursors. The fermentation of sprouted wholemeal by yeast was observed to effectively reduce acrylamide and 5-hydroxymethylfurfural levels due to the consumption of sugars and asparagine during the process (Yıltırak et al., 2021). In contrast, yeast fermentation, which resulted in a 3-unit decrease in pH, reduced acrylamide concentrations but simultaneously increased HMF levels. This effect was attributed to dehydration reactions of reducing sugars, which become predominant under acidic pH conditions. This highlights the dual nature of fermentation, reducing acrylamide but potentially increasing other thermal contaminants, suggesting the need for integrated contaminant profiling during optimization.

### 4.4 Probiotic Interventions

Probiotic consumption is well-established for its safety and various health benefits. The application of probiotic bacteria represents a promising approach for reducing the presence and bioavailability of toxic and harmful substances in vitro (Pop et al., 2022). Consequently, this methodology provides a viable post-processing strategy for lowering acrylamide concentrations in food products (Khorshidian et al., 2020).

It has been found that ingested acrylamide can be broken down by microorganisms, thus contributing to reducing its negative impact on the human body. For example, the probiotic strain *Lactobacillus acidophilus* LA-5, commonly used in yogurt production, can degrade acrylamide, utilizing the ammonia released by amidase as a nitrogen source and the remaining acrylamide or its metabolites as a carbon source (Petka et al., 2021). In addition, another study demonstrated that the acrylamide-degrading ability of *Lactobacillus acidophilus* LA-5 in milk is dependent on the nutritional composition of the environment, being significant only in nutrient-poor environments, in the absence of other readily available sources of nitrogen and carbon (Petka et al., 2022). These results suggest that fermented dairy products, such as yogurt, not only support digestion and maintain gastrointestinal balance, but may also contribute to reducing human exposure to acrylamide.

In addition to the type of food matrix, pH represents another critical factor influencing the effectiveness of probiotic strains in reducing acrylamide (AA) levels. Thus, in an attempt to explore a new post-processing approach for acrylamide reduction, studies have demonstrated that *Lactobacillus acidophilus* LA 45 and *Bifidobacterium longum* ATCC 15707 significantly reduce AA levels, both in food matrices and under simulated digestion conditions. (Choi et al., 2022). Furthermore, the addition of probiotic bacteria to biscuits and chips samples effectively decreases AA levels from 25.35 to 38.54 % at neutral (6.5–7.0) and alkaline pH (10.5–11.0) compared to acidic pH (2.5–3.0), highlighting the importance of tailoring the conditions of use to maximize probiotic potential.

Complex probiotic formulations represent a superior option to the use of a single strain, due to the synergy between strains, the diversity of mechanisms of action, and their ability to adapt and remain stable in different food matrices, thus offering a more effective and versatile solution for reducing food contaminants. However, a recent study in which probiotic formulas were incubated with potato chips and biscuits, followed by an in vitro digestion model, revealed a similar AA reduction to that observed in chemical solutions (Choi et al., 2023). In this study, the synergistic effects between probiotic formulations revealed a strong dependence on specific strains, which raises questions about the generalized applicability of these formulations. For example, the combination of *Lactiplantibacillus plantarum* subsp. *plantarum* ATCC14917 *and Lactobacillus delbrueckii* subsp. *bulgaricus* ATCC11842 recorded the highest reduction of AA (52 %). However, it remains unclear whether these strains could generate similar results in other types of food matrices or under different digestive conditions. The findings highlight the need for further research to clarify the role of each strain in reducing AA and to determine the real potential of probiotic formulations in the detoxification process of this food contaminant.

### 4.5 Use of cations

Alternative treatments involving the addition of cations, notably calcium, have shown effectiveness in mitigating acrylamide formation in bakery products. This reduction is achieved by shifting the Maillard reaction towards caramelization, with divalent cations proving particularly effective (Göncüoğlu Taş et al., 2023). A recent investigation into the use of calcium (CaCl_2_) and magnesium (MgCl_2_) salts for reducing acrylamide formation during the frying of tortilla chips in soybean oil found that 0.12 M MgCl_2_ solutions resulted in a significant 74 % reduction in acrylamide levels, while 0.08 M CaCl_2_ solutions achieved a slightly lower 67 % reduction (Arámbula-Villa et al., 2018). These findings highlight the potential of salt application during masa preparation as a pragmatic and cost-effective method for reducing acrylamide in thermally processed corn-based foods. Similar results were observed in cereal products, where calcium chloride reduced acrylamide by up to 90 % (Kukurová & Ciesarová, 2024). Also, green olives stored for 21 months, when washed before lye treatment, presented the lowest acrylamide levels, with sliced olives containing less acrylamide than other forms, while additives like CaCl_2_ and NaCl increase acrylamide content (Martín-Vertedor et al., 2021). This indicates that both the timing and the method of salt application represent critical variables that may either suppress or facilitate acrylamide formation, depending on the specific processing conditions. Despite the effectiveness of non‑sodium salts in reducing both acrylamide and sodium levels, they also increase the formation of 5-hydroxymethylfurfural and significantly alter the product's volatile profile. This necessitates a meticulous approach in selecting and combining salts to preserve flavor and maintain product quality. Therefore, cation-based mitigation strategies should be evaluated not only for their toxicological efficacy but also for their impact on organoleptic properties and consumer acceptability. While divalent cations treatments offer a viable pathway for acrylamide reduction in cereal- and snack-based foods, their application must be carefully optimized. Future research should aim to balance mitigation efficiency, processing parameters, and sensory outcomes through integrated studies that combine analytical chemistry, sensory analysis, and mechanistic modeling

### 4.6 Application of hydrocolloids

Hydrocolloids, encompassing polysaccharides and proteins, are natural compounds characterized by hydrophilic groups, commonly utilized as food coatings, film-forming materials, and emulsifiers. Due to their ability to form viscous dispersions or gels in aqueous environments during food processing, they have garnered significant interest for their potential role in enhancing food safety (Zhang et al., 2021).

Recent research has highlighted sodium alginic acid as a prominent inhibitor capable of reducing AA and HMF levels by 35.3 % and 26.2 %, respectively (Huang et al., 2022). Furthermore, synergistic effects were observed when alginic acid was combined with ultrasound treatment or blanching, suggesting a possible mechanism involving the disruption of α-dicarbonyl intermediate formation. In another study, hydrocolloid coatings provided more effective in reducing acrylamide content in potato chips compared to aqueous extracts of *Zataria multiflora* and *Allium hirtifolium* indicating economic advantages for use in the production of fried potato crisps (Zokaei et al., 2020). Similar results were observed in French fries, suggesting that coatings likely increased water retention, thereby reducing the Maillard reaction responsible for acrylamide formation and oil uptake (Al-Asmar et al., 2018).

In the context of deep-fried fish nuggets, batters with added hydrocolloids such as chitosan, gum arabic, and xanthan gum showed significant inhibition of acrylamide formation. This effect is attributed to the enhanced moisture retention and reduced oil content, which in turn mitigated acrylamide through diminished Maillard reactions (Jiang et al., 2021). Similarly, in biscuit dough, coatings with gum arabic, pectin, and carboxymethylcellulose also demonstrated significant reduction in acrylamide levels. Notably, gum arabic supplementation preserved the quality and overall consumer acceptability of ammonia biscuits (Mousa, 2019). Furthermore, a “Green Approach” utilizing L-Asparaginase in conjunction with a hydrocolloid protective coating effectively reduced acrylamide levels in potato slices. The application of a zein-pectin nanocomplex resulted in over a 70 % reduction in acrylamide, underscoring the effectiveness of this dual-component system (Bisht et al., 2024).

The inhibitory effects of alginate, pectin, and chitosan on acrylamide formation were studied in fried potato models using heating block and microwave methods. Coating potatoes with alginate, pectin, and chitosan (1 % *w*/*v*) before frying reduced acrylamide formation by 54 %, 51 %, and 41 %, respectively, while alginate and pectin achieved a 5 % reduction under microwave heating (Champrasert et al., 2021). In another study, a series of protein–polysaccharide biopolymer particles were tested as potential acrylamide inhibitors in fried potato strips. The results demonstrated that surface coating with these complex particles, particularly zein–alginate complex particles, effectively mitigates acrylamide formation by reducing the interaction between heat and intermediates (Champrasert et al., 2022). These findings collectively underscore the role of hydrocolloids not only as functional additives but also as active agents in acrylamide reduction. However, further research is required to optimize their concentrations, understand their interactions with diverse food matrices, and evaluate their impact on the bioavailability of nutrients and sensory characteristics of final products. Moreover, the exploration of novel biopolymer combinations and nanostructured delivery systems presents promising directions for next-generation food safety technologies.

## 5 Challenges and future perspectives

The identification of acrylamide is crucial for assessing dietary exposure, particularly in light of its classification by the World Health Organization (WHO) and the Food and Agriculture Organization (FAO), which classify acrylamide as a significant human health concern. This classification is based on acrylamide's capacity to induce cancer in laboratory animals and cause heritable mutations. Thus, assessing acrylamide levels in food is vital for understanding the associated health risks of human consumption.

The effectiveness of acrylamide mitigation strategies depends fundamentally on the sensitivity and specificity of the analytical methods employed. High-performance techniques such as LC-MS/MS and GC–MS are indispensable not only for detecting acrylamide at trace levels in complex food matrices, but also for validating the true efficacy of reduction interventions. These advanced tools allow for the detection of minor but significant decreases in acrylamide concentrations, particularly important in the case of mitigation strategies based on enzymatic treatments or antioxidant incorporation. Without sufficient analytical resolution, these changes may remain undetected, leading to inaccurate conclusions about the success of interventions. At the same time, mitigation strategies must be evaluated beyond their chemical outcomes, taking into account potential technological trade-offs, including undesirable modifications in the sensory characteristics of food products. In this context, analytical precision must be integrated with process design to ensure the development of safe, effective, and consumer-acceptable food safety solutions.

The financial constraints faced by many laboratories worldwide limit routine acrylamide analysis, as the cost of HPLC-MS/MS equipment is prohibitive. Traditional methods like LC-UV and GC-ECD lack the specificity to reliably detect acrylamide in complex food matrices. Furthermore, interferences from compounds such as valine, *N*-acetyl-β-alanine, 3-aminopropionamide, and lactamide challenge even advanced analytical techniques like LC-MS/MS. These observations underscore the necessity for developing more accessible and precise analytical methods for detecting acrylamide. Addressing these challenges will facilitate more accurate assessments of acrylamide levels in food, essential for safeguarding public health.

Given the diversity of analytical techniques available, it is crucial for food industry operators to rigorously assess the levels of AA contaminant, typically present in trace amounts. Exploring environmentally sustainable methods to detect various contaminants is essential for maintaining cost-effectiveness. However, in the absence of advanced equipment that can differentiate overlapping signals, the use of an efficient extraction method becomes pivotal to ensure the reliability of the results. Moreover, traditional quantification methods such as LC-MS and GC–MS, while effective, are expensive and come with notable limitations. Consequently, sensing platforms utilizing various transduction systems have emerged as efficient alternatives for quantifying target molecules in diverse food samples (Rayappa et al., 2021). Future advancements in lab-on-chip applications for detecting and quantifying acrylamide may provide more accessible solutions for this field.

This review underscores significant progress in developing low-acrylamide foods through the incorporation of natural and safe substances. Despite successful reductions in acrylamide levels in specific products such as potato chips and breakfast cereals, overall exposure reductions have been inconsistent. New methods, including air frying and adjustments in home cooking practices, present further opportunities to lower acrylamide exposure.

This review highlights advances in acrylamide mitigation using natural compounds and novel processing techniques. Although significant reductions have been achieved in specific food products, overall dietary exposure remains a concern. By integrating detection sensitivity with mitigation performance, the review emphasizes the need for tailored analytical approaches capable of accurately evaluating reduction strategies across diverse food matrices.

Effective mitigation requires ongoing collaboration among food manufacturers, researchers, and regulatory bodies. Continuous monitoring, coupled with consumer education on acrylamide hazards and healthier cooking methods, is essential. Advances in food processing technologies and ingredient innovations offer promising solutions to minimize acrylamide while preserving food quality. Innovative strategies, such as high-pressure technology, pulsed electric fields, and ultrasound treatments, have shown potential in reducing acrylamide levels. Notably, enhancing enzyme stability through immobilization techniques has proven effective while using magnetic nanoparticles or agarose-based supports. In conclusion, addressing acrylamide in food requires continuous innovation and collaboration to ensure the production of safe and nutritious food.

## CRediT authorship contribution statement

**Ancuţa-Veronica Lupăescu:** Writing – review & editing, Writing – original draft, Visualization, Software, Methodology, Investigation, Formal analysis, Data curation, Conceptualization. **Mircea Oroian:** Visualization, Validation, Supervision, Resources, Project administration, Funding acquisition.

## Funding

This research was supported by ‘Stefan cel Mare’ University of Suceava and by Competitiveness Operational Program–Call: POC/975/1/1/Large infrastructures–Projects for Innovation Clusters, under the contract no. SMIS 2014+ 153837. This work was funded by the Ministry of Research, Innovation and Digitalization within Program 1—Development of national research and development system, Subprogram 1.2—Institutional Performance—RDI excellence funding projects, under contract no. 10PFE/2021.

## Declaration of competing interest

The authors declare that they have no known competing financial interests or personal relationships that could have appeared to influence the work reported in this paper.

## Data Availability

Data will be made available on request.

## References

[bb0005] Aghvami M., Mohammadi A., Khaniki G.J., Ahmadi M., Moazzen M., Arabameri M., Shariatifar N. (2023). Investigation of cocoa and cinnamon effect on acrylamide formation in cakes production using GC/MS method: A risk assessment study. Food Chemistry: X.

[bb0010] Akwagiobe E., Ekpenyong M., Asitok A., Amenaghawon A., Ubi D., Ikharia E., Kusuma H., Antai S. (2023). Strain improvement, artificial intelligence optimization, and sensitivity analysis of asparaginase-mediated acrylamide reduction in sweet potato chips. Journal of Food Science and Technology.

[bb0015] Al-Asmar A., Naviglio D., Giosafatto C.V.L., Mariniello L. (2018). Hydrocolloid-based coatings are effective at reducing acrylamide and oil content of French fries. Coatings.

[bb0020] Alnedhary A.A., Numan A.A., al Hammadi M.M., al Hoded T.H. (2023). Optimization, validation and application of quantitative method for the determination of acrylamide in potato chips samples in Yemen using GC-NPD. Pakistan Journal of Analytical & Environmental Chemistry.

[bb0025] Ameur H., Tlais A.Z.A., Paganoni C., Cozzi S., Suman M., Di Cagno R., Polo A. (2024). Tailor-made fermentation of sourdough reduces the acrylamide content in rye crispbread and improves its sensory and nutritional characteristics. International Journal of Food Microbiology.

[bb0030] Anese M., Suman M. (2013). Mitigation strategies of furan and 5-hydroxymethylfurfural in food. Food Research International.

[bb0035] Arámbula-Villa G., Flores-Casamayor V., Velés-Medina J.J., Salazar R. (2018). Mitigating effect of calcium and magnesium on acrylamide formation in tortilla chips. Cereal Chemistry.

[bb0040] Arisseto A.P., de Figueiredo Toledo M.C., Govaert Y., van Loco J., Fraselle S., Degroodt J.-M. (2008). A modified sample preparation for acrylamide determination in cocoa and coffee products. Food Analytical Methods.

[bb0045] Badawy M.E.I., El-Nouby M.A.M., Kimani P.K., Lim L.W., Rabea E.I. (2022). A review of the modern principles and applications of solid-phase extraction techniques in chromatographic analysis. Analytical Sciences.

[bb0050] Bartkiene E., Jakobsone I., Pugajeva I., Bartkevics V., Zadeike D., Juodeikiene G. (2016). Reducing of acrylamide formation in wheat biscuits supplemented with flaxseed and lupine. LWT.

[bb0055] Bebius A., Reding F., Theurillat V., Konings E., Delatour T., Desmarchelier A. (2024). Determination of acrylamide in coffee, cereals, baby food, cocoa, dry pet food, potato products, vegetable crisps, biscuits, tea, nuts, and spices by LC-MS/MS in a single-laboratory validation: First action 2023.01. Journal of AOAC International.

[bb0060] Becalski A., Lau B.P.-Y., Lewis D., Seaman S.W. (2003). Acrylamide in foods: Occurrence, sources, and modeling. Journal of Agricultural and Food Chemistry.

[bb0065] Bedade D.K., Muley A.B., Singhal R.S. (2019). Magnetic cross-linked enzyme aggregates of acrylamidase from Cupriavidus oxalaticus ICTDB921 for biodegradation of acrylamide from industrial waste water. Bioresource Technology.

[bb0070] Bedade D.K., Singhal R.S. (2017). Isolation and characterization of Acrylamidase from Arthrobacter sp. DBV1 and its ability to biodegrade acrylamide. Applied Biochemistry and Biotechnology.

[bb0075] Bedade D.K., Sutar Y.B., Singhal R.S. (2019). Chitosan coated calcium alginate beads for covalent immobilization of acrylamidase: Process parameters and removal of acrylamide from coffee. Food Chemistry.

[bb0080] Biedermann M., Grundböck F., Fiselier K., Biedermann S., Bürgi C., Grob K. (2010). Acrylamide monitoring in Switzerland, 2007–2009: Results and conclusions. Food Additives & Contaminants: Part A.

[bb0085] Bisht V., Ghosh T., Kumar P., Sharma R., Chamoli S., Patodia H., Navani N.K. (2024). Mitigation of acrylamide in fried food systems using a combination of zein-pectin hydrocolloid complex and a food-grade l-asparaginase. International Journal of Biological Macromolecules.

[bb0090] Borczak B., Sikora M., Kapusta-Duch J., Fołta M., Szewczyk A., Zięć G., Leszczyńska T. (2022). Antioxidative properties and acrylamide content of functional wheat-flour cookies enriched with wild-grown fruits. Molecules.

[bb0095] Brenes-Álvarez M., Ramírez E.M., Brenes M., García-García P., Medina E., Romero C. (2023). New and rapid analytical method using HPLC-MS detection for acrylamide determination in black ripe olives. Foods.

[bib532] Buczek S.B., Cope W.G., McLaughlin R.A., Kwak T.J. (2017). Acute toxicity of polyacrylamide flocculants to early life stages of freshwater mussels. Environmental Toxicology and Chemistry.

[bb0100] Cantrell M.S., McDougal O.M. (2021). Biomedical rationale for acrylamide regulation and methods of detection. Comprehensive Reviews in Food Science and Food Safety.

[bb0105] Champrasert O., Chu J., Meng Q., Viney S., Holmes M., Suwannaporn P., Orfila C. (2021). Inhibitory effect of polysaccharides on acrylamide formation in chemical and food model systems. Food Chemistry.

[bb0110] Champrasert O., Orfila C., Suwannaporn P. (2022). Acrylamide mitigation using zein–polysaccharide complex particles. Food Hydrocolloids.

[bb0115] Chi H., Chen M., Jiao L., Lu Z., Bie X., Zhao H., Lu F. (2021). Characterization of a novel L-Asparaginase from Mycobacterium gordonae with acrylamide mitigation potential. Foods.

[bb0120] Chi H., Jiang Q., Feng Y., Zhang G., Wang Y., Zhu P., Lu F. (2023). Thermal stability enhancement of L-Asparaginase from Corynebacterium glutamicum based on a semi-rational design and its effect on acrylamide mitigation capacity in biscuits. Foods.

[bb0125] Chi H., Zhu X., Shen J., Lu Z., Lu F., Lyu Y., Zhu P. (2023). Thermostability enhancement and insight of L-asparaginase from Mycobacterium sp. via consensus-guided engineering. Applied Microbiology and Biotechnology.

[bb0130] Choi S., Lee Y., Jung M., Kim S., Moon B. (2024). Changes in acrylamide content and quality characteristics of red pepper oil by cooking method and conditions. Food Control.

[bb0135] Choi S.M., Lin H., Xie W., Chu I.K. (2023). Study of potential synergistic effect of probiotic formulas on acrylamide reduction. International Journal of Molecular Sciences.

[bb0140] Choi S.M., Yang L., Chang Y., Chu I.K., Dong N. (2022). Study of the efficacy of probiotic Bacteria to reduce acrylamide in food and in vitro digestion. Foods.

[bb0145] Crawford L.M., Kahlon T.S., Chiu M.M., Wang S.C., Friedman M. (2019). Acrylamide content of experimental and commercial flatbreads. Journal of Food Science.

[bb0150] Curtis T.Y., Halford N.G. (2016). Reducing the acrylamide-forming potential of wheat. Food and Energy Security.

[bb0155] Desmarchelier A., Bebius A., Reding F., Griffin A., Ahijado Fernandez M., Beasley J., Clauzier E., Delatour T. (2022). Towards a consensus LC-MS/MS method for the determination of acrylamide in food that prevents overestimation due to interferences. Food Additives & Contaminants: Part A.

[bb0160] Desmarchelier A., Hamel J., Delatour T. (2020). Sources of overestimation in the analysis of acrylamide-in coffee by liquid chromatography mass spectrometry. Journal of Chromatography A.

[bb0165] Elbashir A.A., Omar M.M.A., Ibrahim W.A.W., Schmitz O.J., Aboul-Enein H.Y. (2014). Acrylamide analysis in food by liquid chromatographic and gas chromatographic methods. Critical Reviews in Analytical Chemistry.

[bb0170] El-Sayed A.A., Abdelhady M.M., Jaafari S.A., Alanazi T.M., Mohammed A.S. (2023). Impact of some enzymatic treatments on acrylamide content in biscuits. Processes.

[bb0175] El-Sayed A.A., El-Maaty S.M.A., Abdelhady M.M. (2023). Acrylamide reduction in potato chips as functional food product via application of enzymes, baker’s yeast, and green tea powder. Scientific African.

[bb0180] Endeshaw H., Belay A. (2020). Optimization of the roasting conditions to lower acrylamide content and improve the nutrient composition and antioxidant properties of Coffea arabica. PLoS One.

[bb0185] España Amórtegui J.C., Ekroth S., Pekar H., Guerrero Dallos J.A. (2024). A green-footprint approach for parallel multiclass analysis of contaminants in roasted coffee via LC-HRMS. Analytical and Bioanalytical Chemistry.

[bb0190] Fan M., Xu X., Lang W., Wang W., Wang X., Xin A., Zhou F., Ding Z., Ye X., Zhu B. (2023). Toxicity, formation, contamination, determination and mitigation of acrylamide in thermally processed plant-based foods and herbal medicines: A review. Ecotoxicology and Environmental Safety.

[bb0195] Feng Y., Shi Y., Huang R., Wang P., Li G. (2023). Simultaneous detection of heterocyclic aromatic amines and acrylamide in thermally processed foods by magnetic solid-phase extraction combined with HPLC-MS/MS based on cysteine-functionalized covalent organic frameworks. Food Chemistry.

[bib531] Gaytán I., Burelo M., Loza-Tavera H. (2021). Current status on the biodegradability of acrylic polymers: microorganisms, enzymes and metabolic pathways involved. Applied Microbiology and Biotechnology.

[bib534] Ghamdi A., Alenezi F., Algoferi M., Alhawas M., Farga A.A., Afifi M. (2020). A review on the new trends of acrylamide toxicity. Biomedical Journal of Scientific & Technical Research.

[bb0200] Göncüoğlu Taş N., Kocadağlı T., Balagiannis D.P., Gökmen V., Parker J.K. (2023). Effect of salts on the formation of acrylamide, 5-hydroxymethylfurfural and flavour compounds in a crust-like glucose/wheat flour dough system during heating. Food Chemistry.

[bb0205] Hamzalıoğlu A., Gökmen V. (2024). Acrylamide in food.

[bb0210] Hasan G.M.M.A., Das A.K., Satter M.A. (2022). Detection of acrylamide traces in some commonly consumed heat-treated carbohydrate-rich foods by GC-MS/MS in Bangladesh. Heliyon.

[bb0215] Huang Y., Li M., Lu J., Hu H., Wang Y., Li C., Huang X., Chen Y., Shen M., Nie S., Xie M. (2022). Inhibitory effect of hydrocolloids and ultrasound treatments on acrylamide and 5-hydroxymethylfurfural formation in French fries. Food Hydrocolloids.

[bib536] Jensen S.N., Hakme E., Feyissa A.H. (2024). Kinetic modeling of acrylamide formation during seaweed bread baking. LWT.

[bb0220] Jiang Y., Qin R., Jia C., Rong J., Hu Y., Liu R. (2021). Hydrocolloid effects on Nε-carboxymethyllysine and acrylamide of deep-fried fish nuggets. Food Bioscience.

[bb0225] Jiao L., Chi H., Lu Z., Zhang C., Chia S.R., Show P.L., Lu F. (2020). Characterization of a novel type I l-asparaginase from Acinetobacter soli and its ability to inhibit acrylamide formation in potato chips. Journal of Bioscience and Bioengineering.

[bb0230] Jing Y., Li X., Hu X., Ma Z., Liu L., Ma X. (2019). Effect of buckwheat extracts on acrylamide formation and the quality of bread. Journal of the Science of Food and Agriculture.

[bb0235] Jozinović A., Šarkanj B., Ačkar Đ., Panak Balentić J., Šubarić D., Cvetković T., Babić J. (2019). Simultaneous determination of acrylamide and Hydroxymethylfurfural in extruded products by LC-MS/MS method. Molecules.

[bb0240] Kamankesh M., Nematollahi A., Mohammadi A., Ferdowsi R. (2021). Investigation of composition, temperature, and heating time in the formation of acrylamide in snack: Central composite design optimization and microextraction coupled with gas chromatography-mass spectrometry. Food Analytical Methods.

[bb0245] Kang C., Ma H., Li Y., Zhang C., Hong Y., Shao M. (2020). Determination of acrylamide in foods by automatic accelerated solvent extraction and gas chromatography-mass spectrometry. Acta Chromatographica.

[bb0250] Karami M., Akbari-Adergani B., Jahed Khaniki G., Shariatifar N., Sadighara P. (2022). Determination and health risk assessment of acrylamide levels in instant coffee products available in Tehran markets by GC-MS. International Journal of Environmental Analytical Chemistry.

[bb0255] Kathuria D., Hamid G., S., & Thakur, A. (2023). Maillard reaction in different food products: Effect on product quality, human health and mitigation strategies. Food Control.

[bb0260] Khaloo Kermani P., Moeenfard M., Farhoosh R., Alves A. (2023). Modified QuEChERS purification method for analysis of acrylamide in roasted Phoenix dactylifera L. seeds via HPLC–PDA. Journal of Food Measurement and Characterization.

[bb0265] Khongsiri C., Ratsamisomsi A., Wilairat P., Tiyapongpattana W. (2024). Low-density solvent-dispersive liquid–liquid microextraction with phase separation by solidification of the aqueous phase for analysis of acrylamide in vegetable and fruit chips by gas chromatography–mass spectrometry. ACS Food Science & Technology.

[bb0270] Khorshidian N., Yousefi M., Shadnoush M., Siadat S.D., Mohammadi M., Mortazavian A.M. (2020). Using probiotics for mitigation of acrylamide in food products: A mini review. Current Opinion in Food Science.

[bb0275] Kukurová K., Ciesarová Z. (2024). Acrylamide in food.

[bb0280] Kulkarni N.H., Muley A.B., Bedade D.K., Singhal R.S. (2020). Cross-linked enzyme aggregates of arylamidase from Cupriavidus oxalaticus ICTDB921: Process optimization, characterization, and application for mitigation of acrylamide in industrial wastewater. Bioprocess and Biosystems Engineering.

[bb0285] Kumari A., Bhattacharya B., Agarwal T., Paul V., Maurya V.K., Chakkaravarthi S., Simal-Gandara J. (2023). Method development and validation for acrylamide in potato cutlet by UHPLC-MS/MS. Food Control.

[bb0290] Ledbetter M., Bartlett L., Fiore A., Montague G., Sturrock K., McNamara G. (2020). Acrylamide in industrial potato crisp manufacturing: A potential tool for its reduction. LWT.

[bb0295] Lodolini E.M., Cabrera-Bañegil M., Fernández A., Delgado-Adámez J., Ramírez R., Martín-Vertedor D. (2019). Monitoring of acrylamide and phenolic compounds in table olive after high hydrostatic pressure and cooking treatments. Food Chemistry.

[bb0300] Lopez-Moreno C., Fernández-Palacios S., Ramírez Márquez P., Ramírez Márquez S.J., Ramírez Montosa C., Carlos Otero J., Ruíz Delgado M.C. (2023). Assessment of acrylamide levels and evaluation of physical attributes in bread made with sourdough and prolonged fermentation. Food Science and Engineering.

[bb0305] Lu X., Chen J., Jiao L., Zhong L., Lu Z., Zhang C., Lu F. (2019). Improvement of the activity of l-asparaginase I improvement of the catalytic activity of l-asparaginase I from bacillus megaterium H-1 by in vitro directed evolution. Journal of Bioscience and Bioengineering.

[bib526] Ma Q., Cai S., Jia Y., Sun X., Yi J., Du J. (2020). Effects of hot-water extract from vine tea (Ampelopsis grossedentata) on acrylamide formation, quality and consumer acceptability of bread. Foods.

[bib529] Martín-Vertedor D., Fernández A., Hernández A., Arias-Calderón R., Delgado-Adámez J., Pérez-Nevado F. (2020). Acrylamide reduction after phenols addition to Californian-style black olives. Food Control.

[bb0310] Martín-Vertedor D., Fernández A., Mesías M., Martínez M., Martín-Tornero E. (2021). Identification of mitigation strategies to reduce acrylamide levels during the production of black olives. Journal of Food Composition and Analysis.

[bib540] Mechi D., Fernández A., Baccouri B., Abaza L., Martín-Vertedor D. (2022). Addition of ‘Chetoui’ olive leaf extract to reduce acrylamide in Californian-style black olive. Food Bioscience.

[bb0315] Mesías M., Morales F.J. (2016). Acrylamide in coffee: Estimation of exposure from vending machines. Journal of Food Composition and Analysis.

[bb0320] Mousa R.M.A. (2019). Simultaneous mitigation of 4(5)-methylimidazole, acrylamide, and 5-hydroxymethylfurfural in ammonia biscuits by supplementing with food hydrocolloids. Food Science & Nutrition.

[bb0325] Nasiri Esfahani B., Kadivar M., Shahedi M., Soleimanian-Zad S. (2017). Reduction of acrylamide in whole-wheat bread by combining lactobacilli and yeast fermentation. Food Additives & Contaminants: Part A.

[bb0330] Negoiță, M., Mihai, A. L., & Horneț, G.-A. (2021). Validation of an analytical method for the determination of acrylamide in potato chips and french fries. The annals of the university dunarea de jos of galati. Fascicle VI - Food Technology, 45(1), 69–85. Doi: 10.35219/foodtechnology.2021.1.05.

[bb0335] Negoiță M., Mihai A.L., Horneț G.A., Duță D.E. (2020). Development of SPE clean-up procedure for acrylamide determination from potato-based products by GC-MS/MS. Open Agriculture.

[bb0340] Nematollahi A., Kamankesh M., Hosseini H., Hadian Z., Ghasemi J., Mohammadi A. (2020). Investigation and determination of acrylamide in 24 types of roasted nuts and seeds using microextraction method coupled with gas chromatography–mass spectrometry: Central composite design. Journal of Food Measurement and Characterization.

[bb0345] Norouzi E., Kamankesh M., Mohammadi A., Attaran A. (2018). Acrylamide in bread samples: Determining using ultrasonic-assisted extraction and microextraction method followed by gas chromatography-mass spectrometry. Journal of Cereal Science.

[bb0350] Nur Hidayah J., Abdul Razis A.F., Jambari N.N., Chai L.C., You L., Sanny M. (2024). Dietary exposure to acrylamide among the Malaysian adult population. Food and Chemical Toxicology.

[bb0355] Onishi Y., Prihanto A.A., Yano S., Takagi K., Umekawa M., Wakayama M. (2015). Effective treatment for suppression of acrylamide formation in fried potato chips using L-asparaginase from Bacillus subtilis. *3*. Biotech.

[bb0360] Oroian M., Amariei S., Gutt G. (2015). Acrylamide in Romanian food using HPLC-UV and a health risk assessment. Food Additives & Contaminants: Part B.

[bib527] Pantalone S., Tonucci L., Cichelli A., Cerretani L., Gómez-Caravaca A.M., D’Alessandro N. (2021). Acrylamide mitigation in processed potato derivatives by addition of natural phenols from olive chain by-products. Journal of Food Composition and Analysis.

[bb0365] Passos C.P., Petronilho S., Serôdio A.F., Neto A.C.M., Torres D., Rudnitskaya A., Coimbra M.A. (2021). HS-SPME gas chromatography approach for Underivatized acrylamide determination in biscuits. Foods.

[bb0370] Pastor K., Ačanski M., Vujić D. (2019). Gas chromatography in food authentication. In Gas chromatography - derivatization, sample preparation, application. IntechOpen.

[bb0375] Pavkovich A.M., Bell D.S. (2018). QuEChERS. In reference module in chemistry. Molecular Sciences and Chemical Engineering.

[bb0380] Petka K., Sroka P., Tarko T., Duda-Chodak A. (2022). The acrylamide degradation by probiotic strain Lactobacillus acidophilus LA-5. Foods.

[bb0385] Petka K., Wajda Ł., Duda-Chodak A. (2021). The utilisation of acrylamide by selected microorganisms used for fermentation of food. Toxics.

[bb0390] Petrarca M.H., Rosa M.A., Queiroz S.C.N., Godoy H.T. (2017). Simultaneous determination of acrylamide and 4-hydroxy-2,5-dimethyl-3(2*H*)-furanone in baby food by liquid chromatography–tandem mass spectrometry. Journal of Chromatography A.

[bb0395] Pop O.L., Suharoschi R., Gabbianelli R. (2022). Biodetoxification and protective properties of probiotics. Microorganisms.

[bb0400] Prata R., Vargas Pérez M., Petrarca M.H., Teixeira Godoy H., Garrido Frenich A., Romero-González R. (2023). Determination of acrylamide in commercial baby foods by LC-QqQ-MS/MS: A simple method for routine analyses. Food Analytical Methods.

[bb0405] Rayappa M.K., Viswanathan P.A., Rattu G., Krishna P.M. (2021). Nanomaterials enabled and bio/chemical analytical sensors for acrylamide detection in thermally processed foods: Advances and outlook. Journal of Agricultural and Food Chemistry.

[bb0410] Roszko M.Ł., Szczepańska M., Szymczyk K., Rzepkowska M. (2020). Dietary risk evaluation of acrylamide intake with bread in Poland, determined by two comparable cleanup procedures. Food Additives & Contaminants: Part B.

[bb0415] Saeed H., Ali H., Soudan H., Embaby A., El-Sharkawy A., Farag A., Ataya F. (2018). Molecular cloning, structural modeling and production of recombinant aspergillus terreus l. asparaginase in Escherichia coli. International Journal of Biological Macromolecules.

[bb0420] Sajed M., Ahmad N., Rashid N. (2022). Temperature dependent autocleavage and applications of recombinant L-asparaginase from Thermococcus kodakarensis for acrylamide mitigation. 3 Biotech.

[bb0425] Saraji M., Javadian S. (2019). Single-drop microextraction combined with gas chromatography-electron capture detection for the determination of acrylamide in food samples. Food Chemistry.

[bb0430] Sarion C., Codină G.G., Dabija A. (2021). Acrylamide in bakery products: A review on health risks, legal regulations and strategies to reduce its formation. International Journal of Environmental Research and Public Health.

[bb0435] Sayah M., Kiarostami V. (2019). Rapid analysis of acrylamide in tap and well water samples by solvent terminated dispersive liquid–liquid microextraction followed by GC–FID. Bulletin of Environmental Contamination and Toxicology.

[bib538] Schouten M.A., Santanatoglia A., Angeloni S., Ricciutelli M., Acquaticci L., Caprioli G., Romani S. (2024). Effects of Nuts, Dried Fruits, Dried Seeds and Black Olives as Enrichment Ingredients on Acrylamide Concentrations in Sweet and Savoury Biscuits. Food and Bioprocess Technology.

[bib537] Schouten M.A., Tappi S., Romani S. (2020). Acrylamide in coffee: formation and possible mitigation strategies–a review. Critical reviews in food science and nutrition.

[bb0440] Seilani F., Shariatifar N., Nazmara S., Khaniki G.J., Sadighara P., Arabameri M. (2021). The analysis and probabilistic health risk assessment of acrylamide level in commercial nuggets samples marketed in Iran: Effect of two different cooking methods. Journal of Environmental Health Science and Engineering.

[bb0445] Shi J., Shao Z., Li H., Zhang Y., Wang S. (2019). Co-extraction and co-purification coupled with HPLC-DAD for simultaneous detection of acrylamide and 5-hydroxymethyl-2-furfural in thermally processed foods. Molecules.

[bb0450] Sun N., Wang Y., Gupta S.K., Rosen C.J. (2020). Potato tuber chemical properties in storage as affected by cultivar and nitrogen rate: Implications for acrylamide formation. Foods.

[bb0455] Sundaram S.S., Kannan A., Chintaluri P.G., Sreekala A.G.V., Nathan V.K. (2024). Thermostable bacterial L-asparaginase for polyacrylamide inhibition and in silico mutational analysis. International Microbiology.

[bb0460] Surma M., Sadowska-Rociek A., Cieślik E., Sznajder-Katarzyńska K. (2017). Optimization of QuEChERS sample preparation method for acrylamide level determination in coffee and coffee substitutes. Microchemical Journal.

[bb0465] Taeymans D., Wood J., Ashby P., Blank I., Studer A., Stadler R.H., O’brien, J., Thompson, S., Silvani, D., & Whitmore, T. (2004). A review of acrylamide: An industry perspective on research, analysis, formation, and control. Critical Reviews in Food Science and Nutrition.

[bib530] Tepe Y., Çebi A. (2019). Acrylamide in Environmental Water: A Review on Sources, Exposure, and Public Health Risks. Exposure and Health.

[bb0470] Timmermann C., Mølck S., Kadawathagedara M., Bjerregaard A., Törnqvist M., Brantsæter A., Pedersen M. (2021). A review of dietary intake of acrylamide in humans. Toxics.

[bib533] Tiwary B.N., Das R., Paul V., Verma P. (2022). Industrial Microbiology and Biotechnology.

[bb0475] Tölgyesi Á., Sharma V.K. (2020). Determination of acrylamide in gingerbread and other food samples by HILIC-MS/MS: A dilute-and-shoot method. Journal of Chromatography B.

[bb0480] Torres J.D., Dueik V., Carré D., Bouchon P. (2019). Effect of the addition of soluble dietary Fiber and green tea polyphenols on acrylamide formation and in vitro starch digestibility in baked starchy matrices. Molecules.

[bb0485] Verma V., Yadav N. (2022). Acrylamide content in starch based commercial foods by using high performance liquid chromatography and its association with browning index. Current Research in Food Science.

[bb0490] Wang J., Cai Z., Zhang N., Hu Z., Zhang J., Ying Y., Zhao Y., Feng L., Zhang J., Wu P. (2022). A novel single step solid-phase extraction combined with bromine derivatization method for rapid determination of acrylamide in coffee and its products by stable isotope dilution ultra-performance liquid chromatography tandem triple quadrupole electrospra. Food Chemistry.

[bb0495] Yeom M.-S., Hwang E.-S. (2020). Quality characteristics, antioxidant activities and acrylamide formation in cookies added with onion peel powder. Korean Journal of Food Preservation.

[bb0500] Yıltırak S., Kocadağlı T., Çelik E.E., Özkaynak Kanmaz E., Gökmen V. (2021). Effects of sprouting and fermentation on free asparagine and reducing sugars in wheat, einkorn, oat, Rye, barley, and buckwheat and on acrylamide and 5-Hydroxymethylfurfural formation during heating. Journal of Agricultural and Food Chemistry.

[bb0505] Yuan B., Ma P., Fan Y., Guan B., Hu Y., Zhang Y., Ni Y. (2022). Construction of L-Asparaginase stable mutation for the application in food acrylamide mitigation. Fermentation.

[bb0510] Zhang N., Zhou Q., Fan D., Xiao J., Zhao Y., Cheng K.-W., Wang M. (2021). Novel roles of hydrocolloids in foods: Inhibition of toxic maillard reaction products formation and attenuation of their harmful effects. Trends in Food Science & Technology.

[bib539] Zhang Y., Jin C., Gökmen V., Mogol B.A. (2024). Acrylamide in Food.

[bb0515] Zhao H., Li N., Li J., Qiao X., Xu Z. (2015). Preparation and application of chitosan-grafted multiwalled carbon nanotubes in matrix solid-phase dispersion extraction for determination of trace acrylamide in foods through high-performance liquid chromatography. Food Analytical Methods.

[bib535] Zhao M., Zhang B., Deng L. (2022). The mechanism of acrylamide-induced neurotoxicity: current status and future perspectives. Frontiers in Nutrition.

[bb0520] Zhou X., Duan M., Gao S., Wang T., Wang Y., Wang X., Zhou Y. (2022). A strategy for reducing acrylamide content in wheat bread by combining acidification rate and prerequisite substance content of Lactobacillus and Saccharomyces cerevisiae. Current Research in Food Science.

[bb0525] Zokaei M., Kamankesh M., Abedi A.-S., Moosavi M.H., Mohammadi A., Rezvani M., Khaneghah A.M. (2020). Reduction in acrylamide formation in potato crisps: Application of extract and hydrocolloid-based coatings. Journal of Food Protection.

